# Diversity and shifts of the bacterial community associated with Baikal sponge mass mortalities

**DOI:** 10.1371/journal.pone.0213926

**Published:** 2019-03-28

**Authors:** Sergei Belikov, Natalia Belkova, Tatiana Butina, Lubov Chernogor, Alexandra Martynova-Van Kley, Armen Nalian, Colin Rorex, Igor Khanaev, Olga Maikova, Sergey Feranchuk

**Affiliations:** 1 Laboratory of Analytical Bioorganic Chemistry, Limnological Institute of Siberian Branch of the Russian Academy of Sciences, Irkutsk, Russia; 2 Department of Biology, Stephen F. Austin State University, Nacogdoches, Texas, United States of America; 3 Department of Informatics, National Research Technical University, Irkutsk, Russia; University of Illinois at Urbana-Champaign, UNITED STATES

## Abstract

The disease of freshwater sponges was first discovered in 2011, when pink samples were found in the Central Basin of Lake Baikal. Subsequently, the visible signs of the disease have changed, and now sponges appear with various symptoms of damage to the body, such as discoloration, tissue necrosis, the formation of brown patches and dirty-purple biofilms on some branches. These signs of the disease are accompanied by the mass death of sponges. We identified differences in microbiomes by sequencing 16S rRNA genes and found changes in the consortium of microorganisms of freshwater Baikal sponges. We found that the observed imbalance in the studied microbial communities of diseased sponges is caused by several different conditionally pathogenic microorganisms that increase their negative effect by acting together and in concert, which leads to the death of photosynthetic microalgae and sponges. Sponges are an important component of coastal communities, and the massive loss of sponges can obviously affect the structure of benthic communities and the purity of water.

## Introduction

Sponges (phylum Porifera) are sessile benthic metazoans and with most belonging to class Demospongiae [1,2]. Sponges are unique filter feeder organisms and serve as efficiently filtering organic particles and plankton from the water column. This ecological function increases water purity and maintains water quality. Most types of sponges are marine; freshwater sponges are much less diverse. All freshwater sponges belong to the suborder Spongillina, consisting of 47 genera with many endemic species [3–5]. Freshwater sponges inhabiting the photic zone habitation are colored in green tones due to symbionts, unicellular green algae or Cyanobacteria [6–10]. Sponges also contain prokaryotic symbionts, which are either an object of nutrition or participate in associative symbiosis with sponges, avoiding the influence of the immune system [11–16]. Thus, a various consortium of bacteria, archaea, algae, fungi, protozoa and viruses and their associative community is structurally defined as a sponge holobiome [17,18].

These microorganisms are able to promote the growth and development of the macro organism, due to the production of regulatory signaling molecules, antibiotics, active metabolites or nutritional components [16,19–22]. Recently, such relationships have recently been actively studied in both marine [23–25] and freshwater sponges [26–30], especially as the response of microbiomes or holobiomes to natural stress (for example, a rise in temperature), and in the context of intrapopulation and biogeographical stability and variability [31–35]. Violation of symbiotic relationships between macro- and microorganisms often leads to diseases and death of sponges, and in the case of the most massive lesions, up to 95% of hosts are affected by the disease [36–38].

An increasing number of reports about sponge diseases and their mass mortality has been published [39–49]. These events can significantly affect the ecosystem as a whole, at least in some cases [36–38,50–54]. Despite the fact that infectious agents are often considered the main factors that cause the mass death of hydrobionts [38–40,48,55,56], only in two cases listed below it has it been possible to isolate and prove the pathogenicity of a certain type of microorganism for marine sponges. In the first case, the strain identified as Alphaproteobacteria *NW4327* was isolated in 1998 from necrotic tissues of the *Rhopaloeides odorabile* sponge, which caused all symptoms of sponge disease in laboratory experiments [55]. Later, the strain was identified as *Pseudoalteromonas agarivorans*, its study showed that it is able to produce collagenase, which destroys the sponge cytoskeleton [57,58]. In the second case, sponge infestation by a consortium of microorganisms in the disease described as a 'sponge necrosis syndrome' affected 30–36% of the sponge Calypsonian (Euplacella) population in the Maldives [48]. Bacterium Rhodobacteraceae and fungus *Rhabdocline* were identified in this consortium and only a combination of these microorganisms caused characteristic signs of disease in the sponges in laboratory experiments, which allowed describing this consortium as an etiological agent.

Numerous hypotheses about the assumed role of microorganisms in the development of marine sponge diseases have been put forward in various studies [41,52,59–64]. Researchers noted significant shifts in the structure of microbial associations, including a decrease in the relative abundance of Proteobacteria and an increase in Bacteroidetes, Firmicutes and Deltaproteobacteria, individual phylotypes of which were found only in infected sponges [41,65,66]. Diseases of marine sponges and corals have been observed for a long time throughout the world, while at the same time there have been no cases of freshwater sponge diseases [36].

The disease of freshwater sponges was first discovered in 2011, when samples of pink color were found in the central basin of Lake Baikal. In subsequent years, the external signs of the disease have changed and from 2013 to the present time, there are sponges with various symptoms of damage to the body, such as discoloration, tissue necrosis, the formation of brown plaque and dirty-violet bacterial covers on separate branches (S1 Video). These signs of the disease are observed especially clearly in the branchy sponge *Lubomirskia baicalensis*, while the cortical and globular sponges have other external signs of the disease. The number of sponges has decreased significantly and now diseased sponges are found throughout Lake Baikal. Disease and mortality of sponges is accompanied by multiple changes in the littoral ecosystem of Lake Baikal [67–72].

The aim of our study is to analyze the changes in the composition of microbiomes in patients and “healthy” freshwater sponges *L*. *baicalensis* collected in the three basins of Lake Baikal in 2015 compared with samples collected in 2010 and 2011. Changes in microbiomes were analyzed by using an approach based on the analysis of sequencing data of the 16S rRNA gene fragments.

## Materials and methods

### Ethics statement

We confirm that the field studies did not involve endangered or protected species. For the described field studies in the water area of Lake Baikal, special permits were not required. Ethical restrictions do not apply to sponges and no permits were required to collect sponge samples.

### Sample collection and treatment

Fresh samples of sponges *L*. *baicalensis* were collected by scuba diving during field trips conducted in 2010, 2011 and 2015 from the southern, central and northern Baikal basins (S1 Fig). The samples were frozen at -20°C immediately after lifting and transported to the laboratory in the refrigerator for subsequent DNA isolation and sequencing analysis. As a control, we used a sample of a healthy sponge (Sp2010healthy) collected in 2010 before the onset of the disease, as well as two samples of 2011, sponge (Sp2011green) without visible symptoms and sick pink sponge (Sp2011pink).

### DNA extraction, PCR amplification, and sequencing

DNA was extracted from the triplicate samples of frozen sponge tissue (0.1–0.2 g) after bead beating using the QIAamp DNA micro Kit (Qiagen Ltd., Crawley, UK) or TRIzol LS reagent (Invitrogen, Ambion, USA) according to the manufacturer’s protocols. Total DNA was suspended in 18μl of RNase free water and stored at—60°C waiting for further analysis. DNA samples of 2015 were transferred to Irkutsk Research Anti-Plague Institute of Siberia and the Far East (Russia) for pyrosequencing on a 454 GS Junior System and samples of 2010 and 2011 were transferred to the RTL Genomics, Lubbock, TX (USA) for high-throughput sequencing on an Illumina platform. Primer sets 357wF/785R and 515yF/926R [73] were used to amplify the variable regions 3–4 and 4–6 of the 16S rRNA gene, using Illumina MiSeq 250 bp chemistry. The universal bacterial primers 518F and 1064R [74] were used to amplify the V4–V6 hypervariable region of the bacterial 16S rRNA gene using the 454 GS Junior sequencing System and with GS FLX Titanium series reagents.

All three sets of primers correspond to the 16S rRNA genes of prokaryotes and chloroplasts, therefore gene fragments of chloroplasts can be amplified and sequenced together with prokaryotes in one experiment. The raw sequencing reads are available under BioProjects ID: PRJNA369024 (454 GS platform) and PRJNA503292 (Illumina MiSeq platform).

### OTU picking

The sequencing reads obtained by the 454 GS technology were preprocessed using Mothur package [75] following conventional setup for filtering unreliable and short oligonucleotides; trim.seq function was applied to raw data files, with parameters ' maxambig = 0, maxhomop = 8, flip = T, bdiffs = 1, pdiffs = 2, qwindowaverage = 35, qwindowsize = 50'). For sequencing reads obtained using Illumina technology, the pipeline of RTL Genomics was used to obtain clean data files. Samples of sponges listed in Table 1 were used in this study.

**Table 1:** Summary of samples used in the survey.

Disease state/ Sample ID	Collection place, near	Group ID	Read archives/ columns in the dataset
**Illumina technology (6 in total):**			
Healthy before disease	Bolshie Koty village (southern basin)	Sp2010healthy	primers 357, 515
Healthy in appearance	Hoboy Cape (central basin)	Sp2011green	primers 357, 515
Diseased	Hoboy Cape (central basin)	Sp2011pink	primers 357, 515 (*)
**454 GS technology (23 in total, collected in 2015 year):**			
**Sponges healthy in appearance:**			
L1,L2,L3,L7,L8	Lystvyanka (southern basin)	Sp2015green	replicates 1–5
OV1,OV3,OV4,OV6	Olkhonskiye Vorota (central basin)		replicates 1–4
T1,T4,T6	Turali Cape (northern basin)		replicates 1–3
**Diseased sponges:**			
L4,L5,L6	Lystvyanka (southern basin)	Sp2015diseased	replicates 1–3
OV2,OV5,OV7,OV8	Olkhonskiye Vorota (central basin)		replicates 1–4
T2,T3,T5,T7	Turali Cape (northern)		replicates 1–4

(*) the similar sample of Sp2011pink/454 was processed and sequenced using the 454 GS technology [69] and included into representations of the dataset in S1 Text.

In the aggregate processing of data files obtained using two different sequencing technologies, an open-reference OTU picking implemented in the QIIME package [76,77] was used. A reference database within the QIIME platform was the database gg_13_5 of Greengenes project compatible with the PICRUSt package [78]. The additional sample of pink sponge (Sp2011pink/454) collected in 2011 and analyzed earlier by Denikina *et al*. [69] was included in the aggregate analysis. This sample was sequenced using the 454 GS technology, and the calculation of the numbers for this additional sample is included in S1 Table for reference. All types of analysis are presented in the “Results” section based on the samples shown in Table 1.

### Downstream analysis

A suite of scripts based on Python and JavaScript were developed for downstream analysis and presentation of results. Tools provided by scikit-bio Python package were used extensively for calculations of biodiversity, PERMANOVA analysis and correspondence analysis. In addition, tools from 'NumPy' and 'SciPy' Python packages were used to expand the approaches used in the study; functions stats.f_oneway and stats.mannwhitneyu from SciPy package were used for ANOVA and ranked tests, stats.kendalltau was used for Kendall correlation. The approaches used for data processing are partly based on the methods described by Feranchuk *et al*. [79]. The functional annotation of microbiomes was implemented using PICRUSt package [78] using a conventional pipeline, as it was described in PICRUSt documentation.

Tools used for data presentation were incorporated into an interactive system developed with the use of d3 JavaScript library running in the front-end and Python scripts running in the back-end. In particular, the stats.f_oneway and stats_mannwhitney Python functions are called in the back-end of the interactive system to present the significant changes of relative abundance in specific phylotypes in two or more groups of samples, following the ANOVA and ranked tests. The heatmap charts represent the estimated significance is represented as–ln (p-value). The source scripts of the interactive system are available at https://github.com/sferanchuk/d3b_charts. A manuscript describing the interactive system for data visualization is under preparation. Finally, the graphics charts generated by the interactive system were manually edited in Inkscape package.

For the additional support of the compatibility between two sequencing technologies, a third-party dataset was combined from the surveys where symbiotic communities of marine sponges were used. Two surveys in the dataset were sequenced with the Illumina (BioProject PRJNA454201) and 454 GS (BioProject Id PRJNA216132) technologies. The dataset was processed with the same pipeline of OTU picking, for a comparative verification of the methodology. The validation of statements about the changes in sponge microbiomes and issues of compatibility between the two technologies are extensively discussed in S1 Text.

## Results

This study focuses on determining the shift in the microbial community of sponges collected in different areas of Lake Baikal in 2015 during the period of their illness and mass death. Differences in microbiomes of sponges were shown using the approach based on the pyrosequencing of the 16S rRNA gene fragment. Data on the composition of the microbiome of one sponge collected before the onset of the disease in 2010, and two samples of sponges collected in 2011, during the appearance of the first symptoms of the disease, were used as a control. These control data were obtained in 2018 using the Illumina sequencing platform. The analysis of the control sample was necessary due to the search for the causes of abrupt changes in the ecosystem of Lake Baikal and the possible relation between the appearance of pink sponges in 2011 and the current state of sponges in 2015. We are very interested in the possible connection between the appearance of pink sponges in 2011 and the current state of sponges in 2015, as well as the composition of microbiomes of a healthy sponge collected before the onset of the disease.

The number of reads included into the OTU table ranged from 1059 (diseased-OV-r3) to 5385 (healthy-T-r3) for the 454 GS samples, and from 11412 (diseased-2011-515) to 61354 (healthy-2010-357) for the Illumina samples. The estimates of sampling depth using Michaelis-Menten fit to rarefaction curves show that in the samples sequenced on Illumina platform the composition of microbiomes at the family level is presented almost completely (underestimate 2.4%). For the 2015 samples of sequenced on 454 GS platform, the average underestimate at the family level is 13.2%.

The challenge of comparative analysis of samples for 3 years was to reduce the biases introduced when the two sequencing technologies were integrated into a single table of abundances. The problems of incompatibility between the two sequencing technologies are well known, as it was extensively demonstrated in Barb *et al*. [80]. However, despite that, the values of abundances for the same group could be drastically different based on the two approaches; the relative changes in abundance within the same method are known to be consistent for several methods [81].

To focus the data processing on a best compatibility between the two sequencing technologies, the closed-reference OTU picking strategy was used, since it results in the most stable taxonomic units [82]. The comparison of samples from several years was mostly presented at high levels of bacterial taxonomy, as it was expected to be more robust to outline the changes in sponge microbiomes. The chloroplast species were intentionally included into the analysis, since unicellular symbiotic algae of *L*. *baicalensis* sponge were considered as an important part of sponge hologenome in a healthy state.

**Fig 1:**
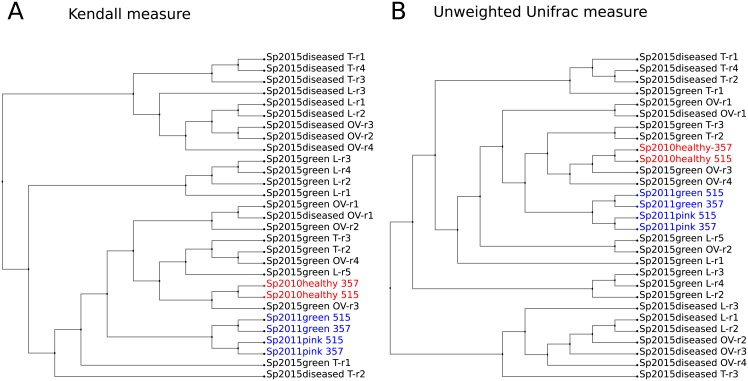
Dendrograms representing a degree of proximity between microbiomes compositions. (A) Rank-based Kendall measure. (B) Presence-based unweighted UniFrac measure. Abundance values in the samples were used at the family level; dendrograms were constructed using UPGMA clustering.

A possible way to compare these data, when the abundance values are biased is to use the presence / absence of a phylotype or to use the phylotype rank, according to Fig 1, which shows dendrograms of proximity between samples. There, the Kendall correlation measure, based on the ranks of the rows, is converted into distances between samples by a simple transformation: distance = 1—correlation (Fig 1A). Unweighted UniFrac measure less clearly separates groups of healthy and sick sponges (Fig 1B).

The proximity between samples estimated by both rank-based and presence-based approaches demonstrates that 2010 healthy sponge is separated from the 2011 samples, and is close to some of the healthy sponges, collected in 2015 in the same area of Baikal near Olkhon Island. A detailed description of the possibility to compare the two technologies is provided in the additional material S1 Text. The consistency of rank comparisons allows us to compare the number of individual families after applying quantile normalization [83].

**Fig 2:**
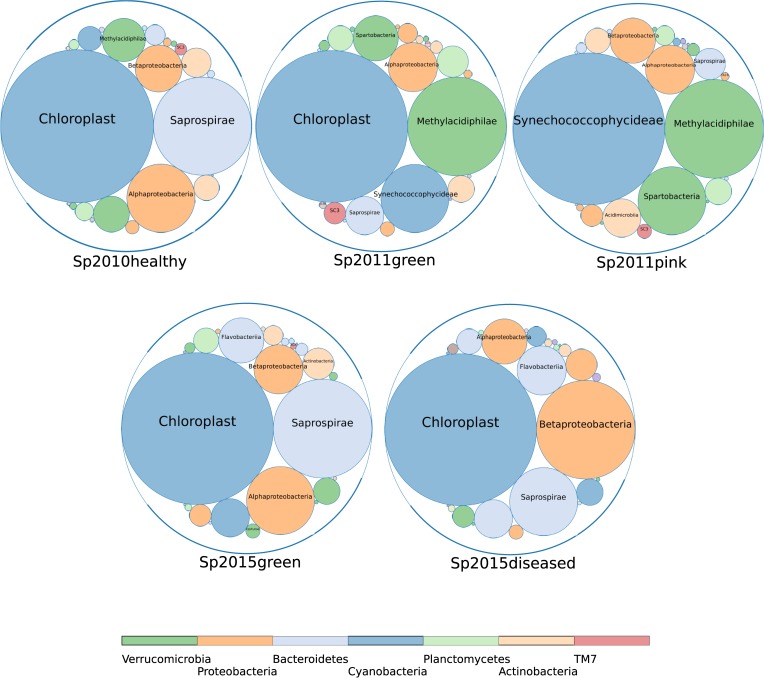
Relative abundances of bacterial groups in the microbiomes of healthy and diseased sponges. Bubble chart is shown at the class level for the 2010, 2011 and 2015 samples of sponges 2010, 2011 and 2015.

Comparison of the composition of microbiomes for samples collected in 2010, 2011 and 2015 is shown in the form of a bubble diagram in Fig 2. The abundance values for this diagram were transformed by quantile normalization in order to reduce the deviations caused by various sequencing technologies. The sizes of the circles have the same proportions in all five charts due to normalization.

Obviously, the healthy in appearance Sp2011green sponge has a high content of Verrucomicrobia (class Methylacidiphilae), which is characteristic of the diseased Sp2011pink sponge. Thus, sponges with no visible signs of the disease may have changes in the microbiomes that are characteristic of sick sponges. Moreover, the content of abundant phylotypes in the microbiome of a healthy sponge Sp2011green has changed dramatically. The increased abundance of Methylacidiphilae is typical only for 2011 sponges, while these Verrucomicrobia are absent in the 2015 sponges. A characteristic feature of Sp2011pink is also the replacement of chloroplasts with cyanobacteria Synechococcophycideae. The most abundant symbionts of healthy sponges of 2010 and 2015 are the phylum Bacteroidetes (Saprospirae / Saprospiria), but they are replaced by Betaproteobacteria in diseased sponges.

**Fig 3:**
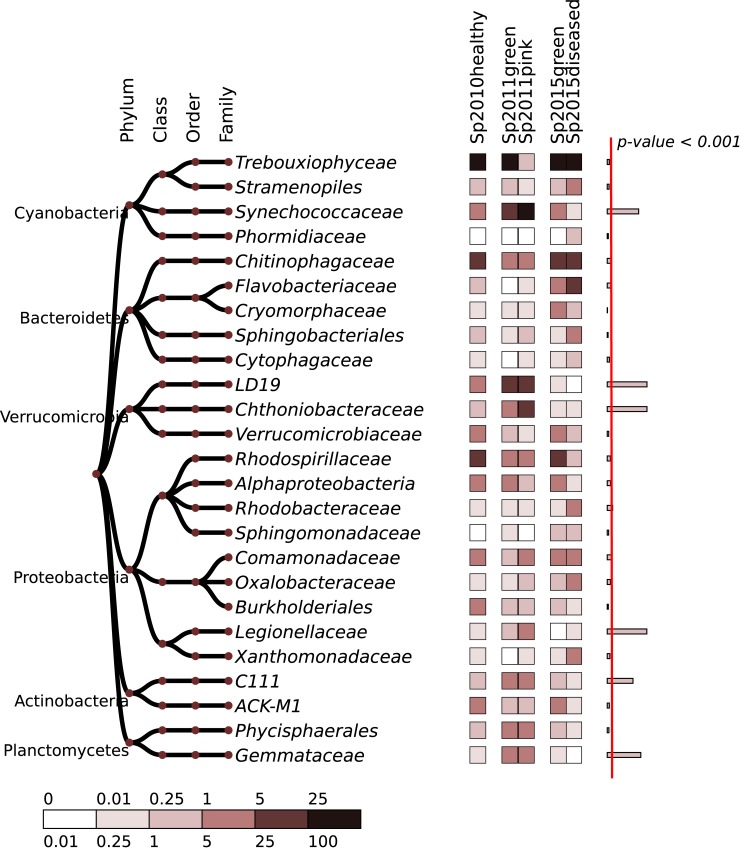
Heatmap of 25 most abundant bacterial families. The relative abundances for individual 2015 samples are averaged. The abundance values were transformed by the quantile normalization, to reduce the biases introduced by sequencing technology. The right column shows a significance of variation between groups, estimated using ANOVA test; width of the bars corresponds to–ln (p-value). Red line separates the significance level of 0.999 (p-value < 0.001).

Similar changes in the number of bacterial groups are shown in heatmap (Fig 3) and in S1 Table. Bacteria abundance values were transformed by normalizing quantiles to reduce the biases introduced by sequencing technology. Evidently, the heterogeneity of microbiomes increases in diseased in sponges, and the average content of chloroplasts in the 2015 sponges does not change significantly.

**Fig 4:**
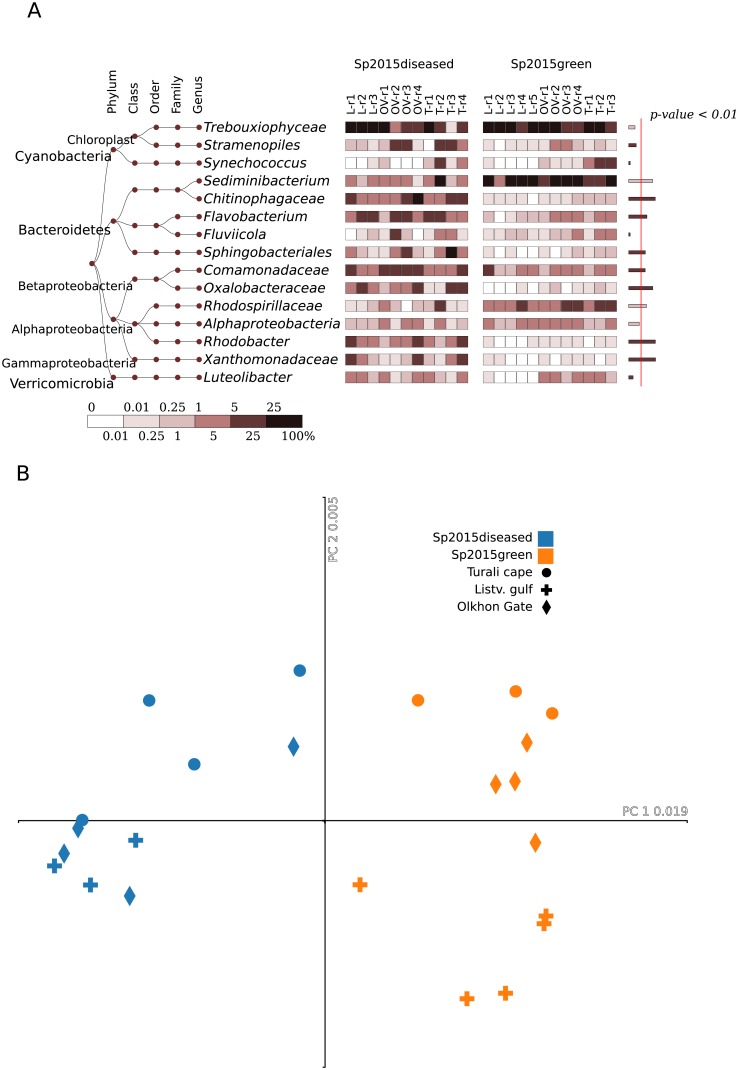
A comparative presentation of the 2015 samples. Result given for (A) heatmap of the 15 most abundant bacterial groups, at the genus level. The right column on the right shows a significant difference between the healthy and diseased samples, estimated using Mann-Whitney test; width of the bars corresponds to–ln (p-value). Red line separates the significance level of 0.99 (p-value < 0.01). (B) Correspondence analysis at the OTU level based on the unweighted UniFrac measure as a distance.

Combining data on the composition of microbiomes in the groups of healthy and sick sponges of 2015 is not correct, since significant changes are observed in individual samples, and the number of bacteria varies randomly between sponge samples (Fig 4, S2 Fig).

The differences in the composition of microbiomes are observed in more detailed taxonomic annotations and in the distribution of the minor components of the microbiome. At the genus level, many unclassified Xanthomonadaceae, Comamonadaceae, Oxalobacteraceae, Chitinophagaceae, Flavobacterium and Rhodobacter should be distinguished in diseased sponges (Fig 4A). The numbers of these groups and OTU in these groups vary from sample to sample, and no single bacterium can be closely associated with signs of disease.

The distribution of chloroplasts in the samples indicates that unicellular symbiotic algae are an important component of sponge microbiomes, but the reasons for such drastic changes remain unknown and require further study. The abundance of chloroplasts contributes to the uniformity of the microbiomes, but the average proportions of the microbial species that separate the healthy and diseased sponge remain almost the same even without taking into account chloroplasts, as explained in S1 Text.

The dependence of microbiomes on the condition of sponge disease is also clearly seen in the graph of compliance analysis (Fig 4B). There is a separation of samples by geographic location, but the separation of sponges for health reasons of sponges is more significant. Estimates of species richness based on the direct amount of OTU and extrapolation of Chao and Ace indices show that the number of species in Northern Baikal (Turali Cape) is greater than in the regions of Southern Baikal, where the anthropogenic load is higher (p-value <0.003 for Ace index) (S2 Table).

**Fig 5:**
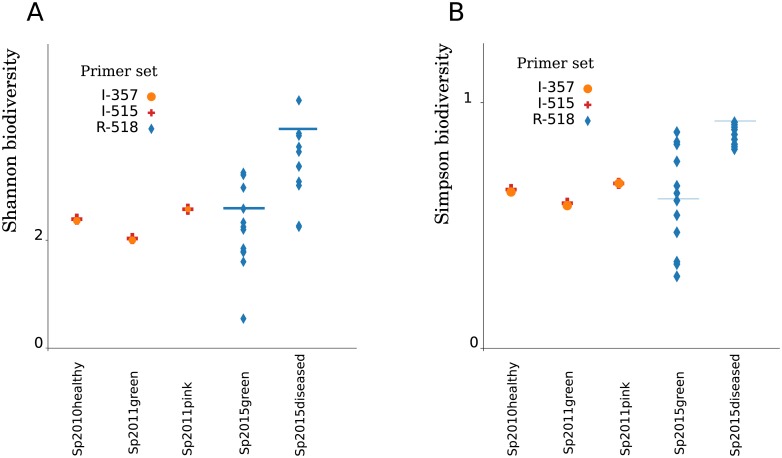
An increase in heterogeneity of diseased samples. Biodiversity values for the samples used in the study (A) Shannon distribution and (B) Simpson distribution. Bars indicate the values for a composition of aggregated communities.

Shannon's biological diversity increases in diseased sponges of 2015 even more than in 2011 (Fig 5). Both Shannon (Fig 5A) and Simpson (Fig 5B) estimates of biodiversity are indicators of community unevenness; hence, microbiomes of diseased sponges are more heterogeneous and that dysbiosis increases with the development of the disease.

We evaluated at a first approximation the properties of a dysbiotic state in diseased and relatively healthy sponges by a functional annotation of functional annotation of relative changes in metabolic pathways. S2 Fig shows the heat map with the results of functional annotation. We have not identified an increase in carbon fixation, methane and nitrogen metabolism and a decrease in the biosynthesis of antibiotics have been noted. A detailed analysis of food chains of microbial communities developing in dying sponges is beyond the scope of this study, but requires a detailed consideration.

## Discussion

Lake Baikal is the deepest freshwater lake in the world. It has a volume of 23,000 km^3^, a depth of 1,637 m and an age exceeding 25 million years [84,85]. Endemic freshwater Baikal sponges of the Lubomirskiidae family dominate the littoral zone of the Lake and their biomass is more than 700 g/m^2^ [86–88]. This sponge biomass is unusually high for freshwater body [89], but it is comparable to coastal Antarctic benthic communities [90] and some reefs [91].

According to the current systematics, the family Lubomirskiidae of endemic sponges in Lake Baikal is represented by 4 genera (*Lubomirskia*, *Baikalospongia*, *Rezinkovia* and *Swartschewskia*) and by 14 species [92,93]. Baikal sponges inhabit depths from one meter down to the maximum depth, but they are most concentrated at depths between 5 and 40 meters, where their biomass exceeds all other benthic organisms taken together [86,87].

Healthy sponges *L*. *baicalensis* sponges have a saturated green color due to the presence of a large number of symbiotic green alga *Choricystis* sp. (Trebouxiophyceae). Disease of freshwater Baikal sponges was recorded for the first time in 2011 by Bormotov [67], and it was accompanied by the change of green color into pink. No other signs of the disease, such as a change in the consistency of the spongin or tissue necrosis were registered. *L*. *baicalensis* is the most susceptible to the disease. It is distributed throughout the lake at depths from 1 to 50 m and the most numerous between depths of 5 and 20 meters [71,94]. Sponges with abnormal coloring were found only in the central basin of Baikal at depths of 25–55 meters (S1 Fig). This distribution of pink sponges calls into question some of the hypotheses about the possible causes of the sponge disease, such as global warming and the anthropogenic impact. In these places, the anthropogenic impact is minimal, and large depths determine the constant water temperature [68,71].

The comparisons of the microbial community of sponges revealed that the symbiotic algae *Choricystis* sp. (class Trebouxiophyceae) is completely absent in the Sp2011pink and is replaced by the cyanobacterium Synechococcaceae (Fig 2). The Synechococcaceae are the family of Cyanobacteria and are typical picoplanktonic Cyanoprokaryota in the littoral and deep-water areas of Lake Baikal [95].

Together with the appearance of Cyanobacteria in the pink sponge, there was a relative abundance of the bacteria of the family Chthoniobacteraceae and *LD19* (phylum Verrucomicrobia) as well as of the minor families *C111* (Actinobacteria), Legionellaceae (Gammaproteobacteria), Gemmataceae and Phycisphaerales order (Planctomycetes) had increased (Fig 3, S1 Table). A similar increase in the relative abundance of the same family of bacteria is found in Sp2011green without visible signs of the disease. Thus, sponges with the absence of visible signs of the disease may have changes in microbiomes typical for diseased sponges. The principal (core) microbiome of a healthy sponge has changed significantly, and the relative content of Chitinophagaceae, Rhodospirillacea and Comamonadaceae which was most abundant in the healthy sponge Sp2010healthy, has decreased dramatically.

In order to identify a possible relation between the sponge disease and differences in habitats, we collected samples of healthy and diseased sponges in the southern, central and northern basins of Baikal, in which the anthropogenic load decreases from south to north. We found that the number of the most abundant phyla in diseased and healthy sponges did not differ substantially (Fig 2). In addition, the composition of bacteria in sponge samples near Listvyanka, where a high concentration of biogenic elements was detected [71], is very similar to the composition of sponge microbiomes from cleaner areas of Baikal. The difference between these locations could be detected only by the absence of several minor components of microbiomes in the samples from Listvyanka. At the same time, the mortality of sponges in this region is the highest, which apparently indicates a lack of a direct correlation between the composition of microbiomes and the mortality of sponges.

Changes in the bacterial composition of sponges in 2015 differ significantly from changes in the sponge microbiomes in 2011. The pink sponge microbial samples collected in 2011 differ from the healthy sponge microbiome collected in 2010 by replacing the chloroplast with the cyanobacterium *Synechococcu*s sp. and the emergence of uncultivated Verrucomicrobia (Chthoniobacteraceae and LD19). Previously, we assumed that an increase in Verrucomicrobia *LD19* might be associated with an increased concentration of methane in the Baikal water [69].

We found that Verrucomicrobia LD19 and Chthoniobacteraceae are absent in the sponge microbiomes of 2015, in contrast to 2011, as was shown previously [69]. A decrease in methane concentration was not noted in the 2015 samples, which indicates that our assumption was erroneous. In addition, Verrucomicrobia *LD19* does not contain genes for methane monooxygenase and, therefore it probably cannot oxidize methane, unlike its phylogenetically closely related acidophilic methanotroph *Methylacidiphilum infernorum* [96]. In addition, in the diseased sponges of year 2015, the relative abundance of Chthoniobacteraceae was significantly lower.

However, “healthy or green” sponges of 2015, with no visible signs of disease, have significant shifts in the microbiomes compared to Sp2010healthy (Fig 5), although the content of chloroplast is high in most samples. The relative abundance of Trebouxiophyceae, Procabacteraceae and unclassified Alphaproteobacteria is lower in sponges of 2015, but the content of Rhodobacteraceae, Comamonadaceae, Flavobacteriaceae, Oxalobacteraceae, Xanthomonacacaces and others has increased (Fig 4A, S1 Table). These changes in the composition of microbiomes are distributed randomly in individual samples of sponges collected in 2015 (Fig 4A, S2 Fig). The representatives of *Flavobacterium*, Comamonadaceae and Rhodobacteraceae have been observed to have adaptive survival strategies and transformations to opportunistic pathogens [97–99]. This suggests that the sponges in the diseased state are suffering the most from opportunistic pathogens of different origins.

Most of the bacteria, whose abundance has grown in diseased sponges, are Bacteroidetes and Proteobacteria, many of which may have quorum sensing activity, that affecting pathogenicity and virulence factors, as well as the ability to form biofilms [100–102]. The presence of QS activity can probably lead to the coordinated joint action of several opportunistic pathogens and may be the cause of the rapid death of the Baikal sponges. Although this relationship between the QS activity in bacteria and disease of sponges is not known, it has been described in corals infected with the white band disease [103,104].

Thus, our research shows that sponge diseases in 2011 and 2015 are fundamentally different, as shown by the PERMANOVA statistical tests described in S1 Text.

A common sign uniting the events of 2011 and 2015 is the inhibition of the growth of symbiotic green algae. Based on this data, we can assume two different hypotheses for the development of the disease. In the first scenario, an unknown aggressive factor arose in the central basin of Lake Baikal, which led to the rapid and complete destruction of green symbionts and the subsequent dying off sponges. Then the concentration of this factor decreased due to the mixing with water, which reduced its aggressive effect and led to a partial, rather than complete, inhibition of the development of symbiotic algae in sponges. On the second hypotheses, from 2013 to the present time, another factor (quorum sensing inducer?) came into the game.

This may cause significant inhibition of the immune system of the sponges and can triggers the development of coordinated action of opportunistic pathogens. Baikal sponge death in 2014–2018 is explained mainly by the development of opportunistic pathogens, which abundance differ from sample to sample. Determining the cause of the disease and the study activities of quorum sensing and quorum quenching in the diseased and convalescent sponges will be the goal of our further research.

## Conclusions

We considered the possibility of using various technologies for sequencing microbiomes of diseased and healthy Baikal sponges. This study allowed us to expand our understanding of changes in the consortium of microorganisms and their complex symbiotic relationships in freshwater Baikal sponges. We found large-scale changes in the microflora of Baikal sponges and an increase in the number of Bacteroidetes and Proteobacteria, and found several opportunistic microorganisms in diseased sponges, which probably act in concert. Understanding how diseases arise and spread in freshwater sponges, and finding their etiological agents, is key to supporting healthy aquatic biodiversity in the Lake Baikal ecosystem.

## Supporting information

S1 Video Underwater surveys of sponges before and during the crisis. (MP4)

S1 Text The validation of bioinformatics pipeline. Relative abundancies of microbial groups and degree of separation between healthy and diseased samples are evaluated using two independent sequencing experiments for a sample of pink sponge collected in 2011.
(PDF)

S1 Fig Places of detection and collection of sponges. Four pointed stars—places of detection of pink sponges in 2011; 5 pointed stars—places for collecting samples of healthy and sick sponges in 2015. 1—cape Hoboy, July 2011, 30–45 meters; 2 –cape Orso, July 2011, 30–45 meters; 3 –cape Ukhan, October 2011, 25–35 meters; 4– cape Izhimey, November 2011, 45–55 meters; 5 –Ushkany Islands, November 2011, 25–35 meters.
(TIF)

S2 Fig Changes in metabolic pathways of diseased sponges. The relative presence of functional groups in microbiomes of sponges from 2015 are presented as heatmap chart, in units of Z-score, for 'Energy metabolism' and 'Biosynthesis of Other Secondary Metabolites' KEGG ontology terms.
(TIF)

S1 Table Abundance counts of microbiomes in the survey, at a level of genus, for 100 most abundant taxonomic phylotypes. (XLS)

S2 Table Values for alpha-diversity and estimates of significance for differences between diversity indices. (PDF)
