# Supplementary material for: Diversity and shifts of the bacterial community associated with Baikal sponge mass mortalities
Source: PLoS One. 2019 Mar 28;14(3):e0213926. doi: 10.1371/journal.pone.0213926 (PMC6438488; doi:10.1371/journal.pone.0213926)
Supplement: S1 Text — Relative abundancies of microbial groups and degree of separation between healthy and diseased samples are evaluated using two independent sequencing experiments for a sample of pink sponge collected in 2011. (PDF) [file pone.0213926.s002.pdf]

S1 Text. The validation of methodology

Supplementary material to the study:

Diversity and shifts of the bacterial community associated with Baikal sponge mass mortalities

authored by

Sergei Belikov, Natalia Belkova, Tatiana Butina, Lubov Chernogor, Alexandra Martynova-Van Kley, Armen Nalian, Colin Rorex, Igor Khanaev, Olga Maikova, Sergey Feranchuk

The results of the statistical analysis are confirmed by the conclusions briefly listed below:

1. The applicability of rank-based and presence-based comparisons between samples sequenced by different technologies;
2. Increase of diversity and heterogeneity in samples collected in 2015;
3. The separation of samples collected in 2010, 2011 and 2015 by overall composition of microbiomes;
4. The compatibility of relative abundancies between two sequenced technologies for specific phylotypes after applying quantile normalization;
5. The separation between healthy and diseased samples in 2015 by overall composition of microbiomes and by abundance of some bacterial groups;
6. The presence of Chloroplast in the presented analysis does not prevent the assessment of the heterogeneity of bacterial species and their relative abundance.

Rarefaction curves, for samples obtained by different technologies shown in S1_fig. The samples obtained by 454 technology are somewhat underrepresented, at average at 13% by Michaelis-Menten fit. The samples by Illumina technology are almost saturated, as it is natural due to higher sequencing depth. Shannon's diversity is relatively high for the 2015 samples (Fig 3 in the main text), and the observed separation between healthy and sick samples suggests that this measure is effective for describing the distribution of the number of the ecosystem being studied, despite the observed incomplete coverage. In addition, as the estimated Shannon diversity is relatively low for samples of 2010 and 2011 year, with sufficient coverage, rarefying this sample to level comparable to the 2015 samples will not significantly affect Shannon's diversity values and other integral properties of population size distributions. Therefore, the heterogeneity of sponges microbiomes did indeed increased in 2015 year relatively to previous observations.

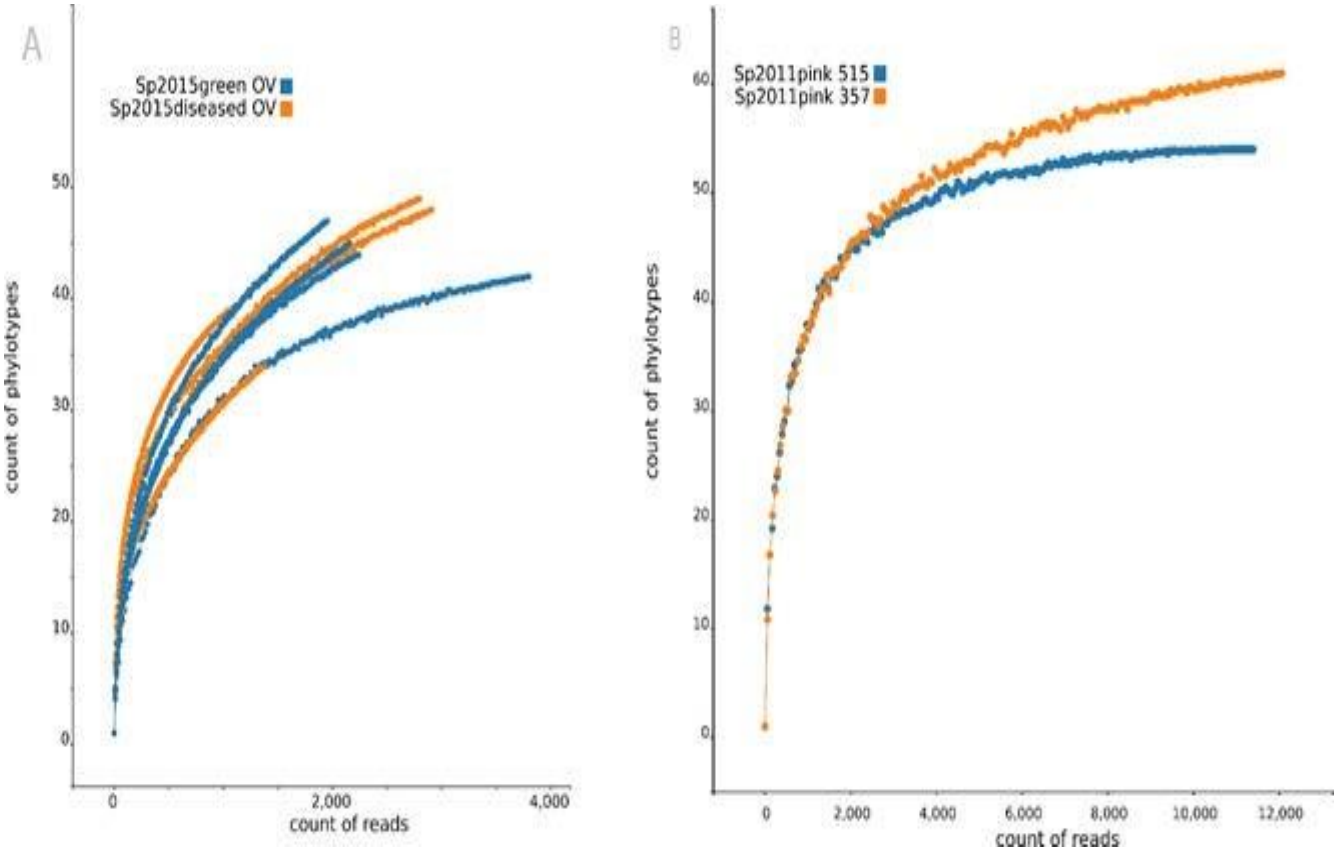

S1_fig. Rarefaction curves, at the level of family. Results for (A) 7 samples from Olkhon Vorota area collected in 2015 and sequenced using 454 technology. (B) Samples from 2011 year, collected near Olkhon Island, amplified by two primer pairs and sequenced using Illumina technology.

The values of abundances are known to be incomparable for data obtained by two technologies. The other ways to compare that data are to use presence/absence of some phylotype or to use rank of phylotype for comparison. These ways indeed performed well as shown below.

Comparison between several measures of beta-diversity presented in S1 Table, in order to suggest in which way the values of abundances for two technologies could be most compatible. Significance of separations between groups of samples for healthy and diseased sponges shown in units of p-value. Matrix of distances between the samples was processed using PERMANOVA approach with 4000 iterations to get the p-value. Distances between samples were estimated from values of relative abundances, reduced to a level of family in taxonomy. Several metrics used to calculate the distances as shown in S1 Table.

S1 Table. The results of PERMANOVA function implemented in scikit-bio package.

Measure	Open-ref (*)	Closed-ref	Closed-ref without Chloroplast
Pearson	0.00925	0.0065	0.0005
Kendall	0.00025	0.00025	0.00025
Spearman	0.00775	0.00725	0.00075
Euclidean	0.003	0.002	0.00025
Bray-Curtis	0.00025	0.002	0.0005
Jaccard	0.00025	0.00025	0.00025
Morisita-Horn	0.0055	0.00275	0.00025
UniFrac-unweighted	0.00025	0.00025	0.00025
UniFrac-weighted	0.00175	0.00175	0.00025

(*) the results estimated in p-value for QIIME open-reference OTU picking for the same dataset

In can be seen that UniFrac-unweighted measure and Jackard measure (S1 Table) which are based on the comparison of presence/absence of the phylotypes, are effective in separation of samples to healthy and diseased groups, when both groups contain samples obtained by two technologies. A Kendall measure of correlation, which is based on ranks of rows, could be transformed to the distances between samples just by a simple transformation: *distance = 1 - correlation.* It is also effective as measure of distance in separation of samples. The results shown in S1 Table support choice of closed-reference OTU picking strategy accepted in the study, as it provides more stable and consistent separation of samples, than the open-reference OTU picking.

Even the raw values of abundance allow comparing visually the compositions of microbiomes in sponges for data obtained by different sequencing methods S2_fig.

A

B

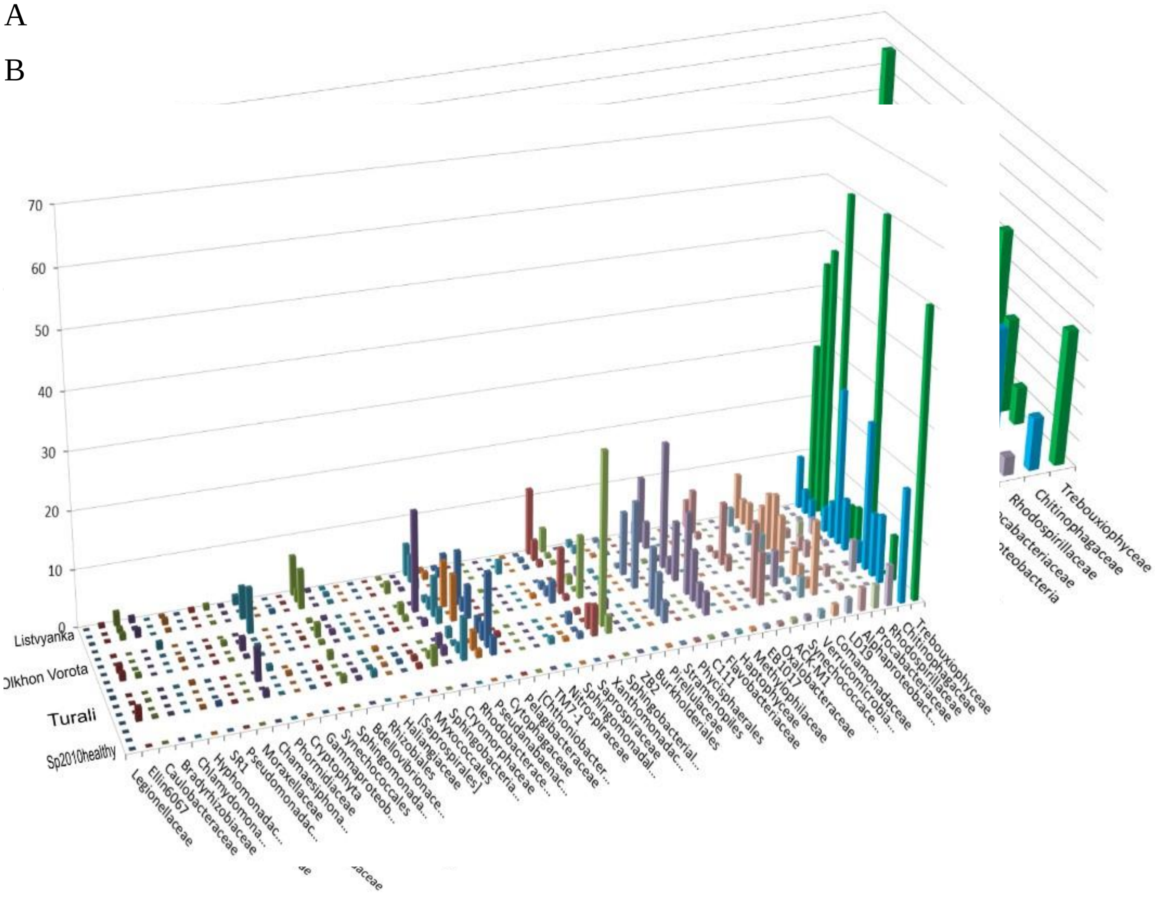
70
60
50
40
30
20
10
0
Listvyanka
lkhon Vorota
Turali
Sp2010healthy
Legionellaceae
Ellin6067
Caulobacteraceae
Bradyrhizobiaceae
Chlamydomona...
Hyphomonadac...
SR1
Pseudomonadac...
Moraxellaceae
Chamaesiphona...
Phormidiaceae
Cryptophyta
Gammaproteob...
Synechococcales
Sphingomonada...
Bdellovibrionace...
Rhizobiales
Haliangiaceae
[Saprospirales]
Myxococcales
Sphingobacteria...
Cryomorphaceae
Rhodobacterace...
Pseudanabaenac...
Cytophagaceae
Pelagibacteraceae
[Chthoniobacter...
TM7-1
Nitrospiraceae
Sphingomonadal...
Saprospiraceae
Xanthomonadac...
ZB2
Sphingobacterial...
Burkholderiales
Pirellulaceae
Stramenopiles
Phycisphaerales
C111
Flavobacteriaceae
Haptophyceae
Methylophilaceae
EB1017
Oxalobacteraceae
ACK-M1
Synechococcace...
Verrucomicrobia...
Comamonadaceae
LD19
Alphaproteobact...
Procabacteriaceae
Rhodospirillaceae
Chitinophagaceae
Trebouxiophyceae
Rhodospirillaceae
Chitinophagaceae
Trebouxiophyceae

C

S2_ fig. **Composition of healthy sponges microbiomes.** (A) Healthy samples of 2015 sequenced by 454 and control Sp2010healthy sequenced by Illumina. (B) Diseased samples of 2015 sequenced by 454 and control Sp2010healthy sequenced by Illumina. (C) Comparison of abundances in microbiomes of samples Sp2011-pink Sp2011green and Sp2010heathy sequenced by Illumina.

Dendrograms degree of proximity of microbiome compositions depending at the measurement method used presented in S3_fig. The composition of dominant species shown to be similar for the two sequencing methods, but Kendall measure more clearly separates groups of healthy and diseased sponges (S3A_fig), as opposed to unweighed UniFrac measure (S3B_fig). Shown, that the 2011 sponges are definitely different from Sp2010healthy sponges. The microbiome composition of the healthy Sp2015green OV-r3 sponge, isolated in 2015, is close to the 2010 sponge microbiome, despite the use of various sequencing methods. The same division into groups is observed in S2_fig.

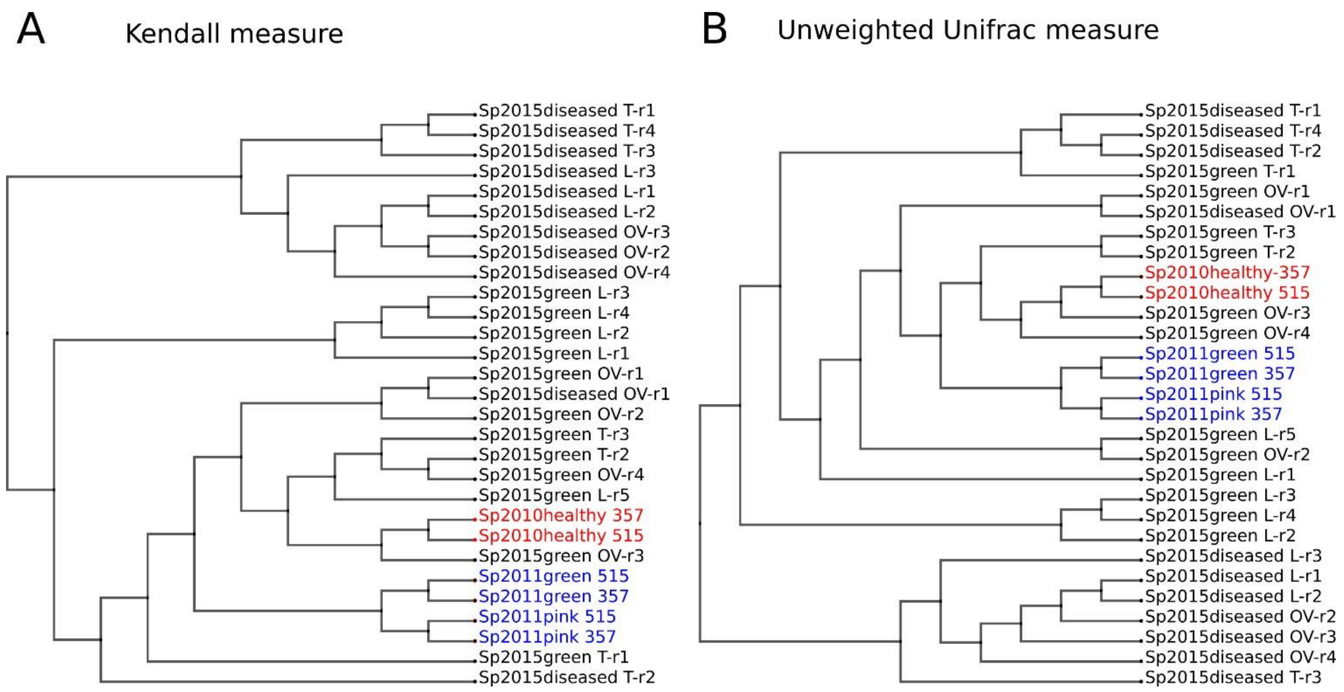

S3_fig. Dendrograms representing the degree of proximity between microbiome compositions. (A) Rank-based Kendall measure. (B) unweighed UniFrac measure. Abundance values in the samples were used at a level of family; dendrograms were constructed using UPGMA clustering.

But to get more rigorous comparison of relative abundance of species in two periods, the quantile normalization could be applied to table of abundances composed from both periods. In the

technique of quantile normalization, only a rank of each species is used to get the transformed values of abundances. The good performance of Kendall correlation as a distance measure suggests that the quantile transformation should improve the consistency between values of abundances.

The data for the same samples Sp2011pink, sequenced by two different technologies using three pairs of primers are presented in S4_fig. The previously published data on high levels of Cyanobacteria and Verrucomicrobia in Sp2011pink are confirmed even for non-transformed values for both types of sequencing. However, the incompatibility of numbers between the 454 and Illumina technologies is clearly visible in the top row. Nevertheless, the distribution of phylotypes in samples looks much more comparable after applying quantile normalization.

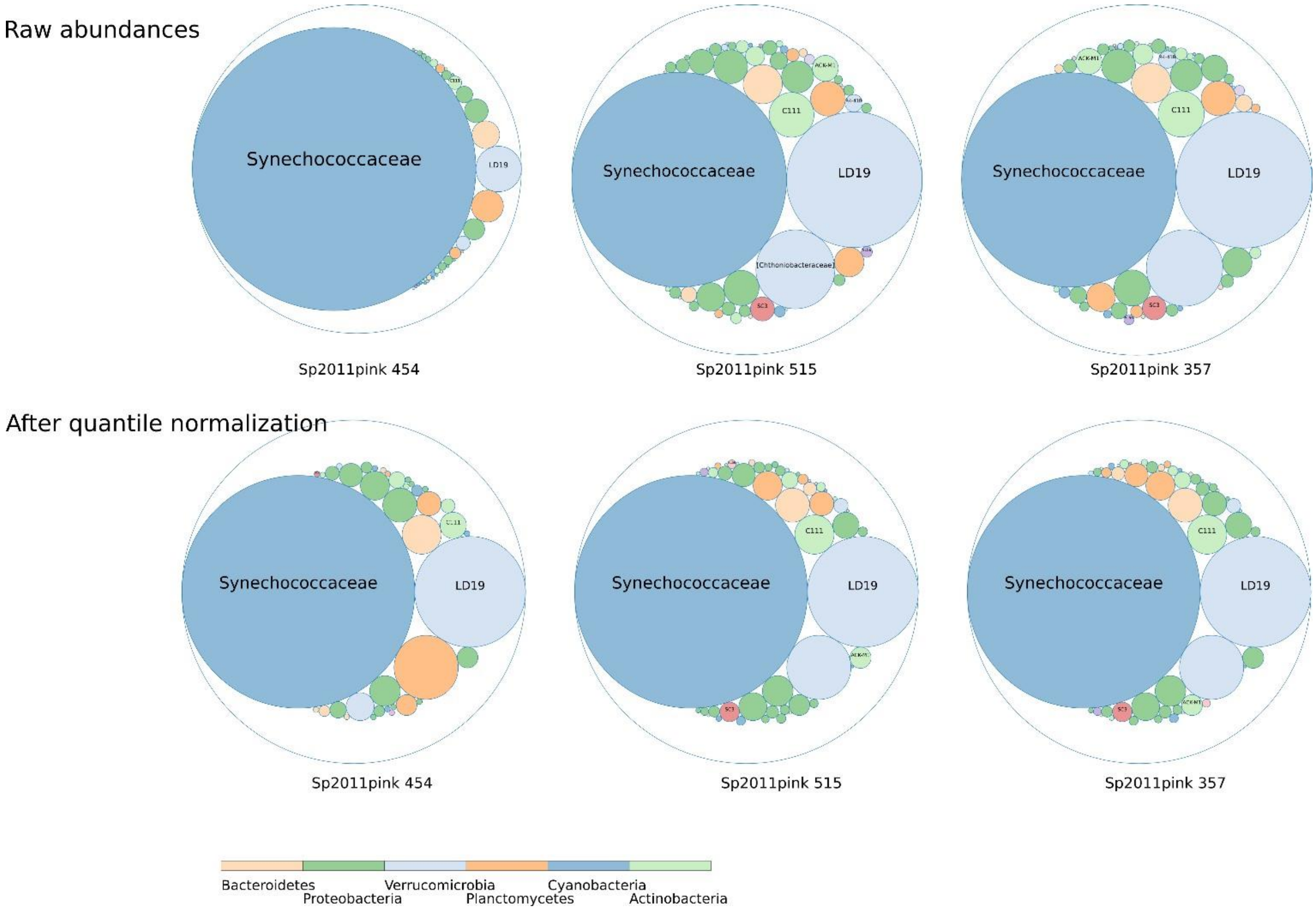

S4_fig. Bubble chart representing the influence of quantile normalization. Data obtained using three technologies for the same sample of pink sponge collected in 2011 before and after quantile normalization.

To study a contribution of chloroplast species to a distribution of relative abundances, several tests performed, as shown below. These trees demonstrate that the distribution of samples is similar,

whatever abundances for Chloroplasts taken into account or not (S5_Fig). Samples of 2010 and 2011 are in any case divided into both trees, which supports the hypothesis about the time of onset of the disease.

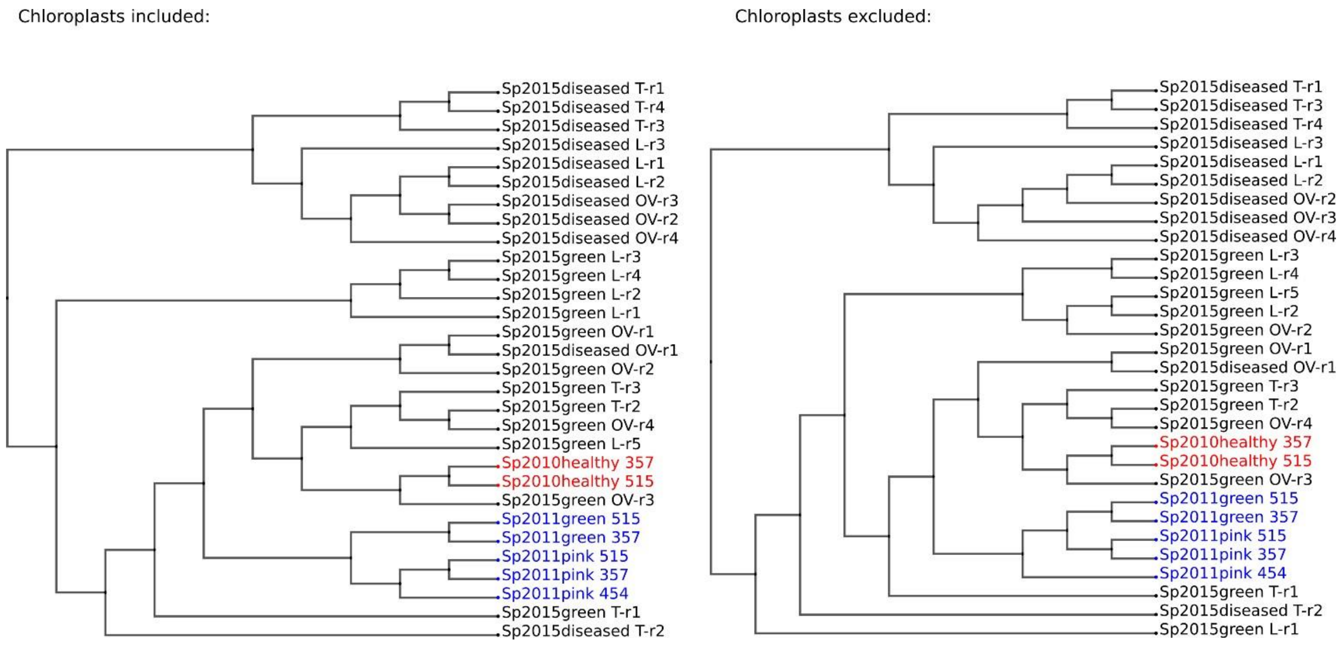

S5_fig. Dendrograms, constructed using a ranked Kendall measure. Influence of presence or absence of Chloroplasts on proximity of the microbiomes composition: (A) Complete data. (B) Chloroplasts are excluded. The dendrograms constructed using UPGMA clustering; abundance in the samples used at the family level.

The separation between healthy (green) and diseased samples is also significant in both cases, Shannon index is higher in diseased samples, p-value = 0.001 on a level of family (S2 Table). The bacterial phylotypes specific to diseased samples and to healthy samples (S6_fig) in both cases are also identified with a consistence to results shown in Fig 4 (main text). Chloroplast species contribute to relative abundance values, so the list of the most common phylotypes differs on two heatmaps (S6_fig). However, this does not affect the observation of changes in microbial composition of the sponge samples discussed in the study.

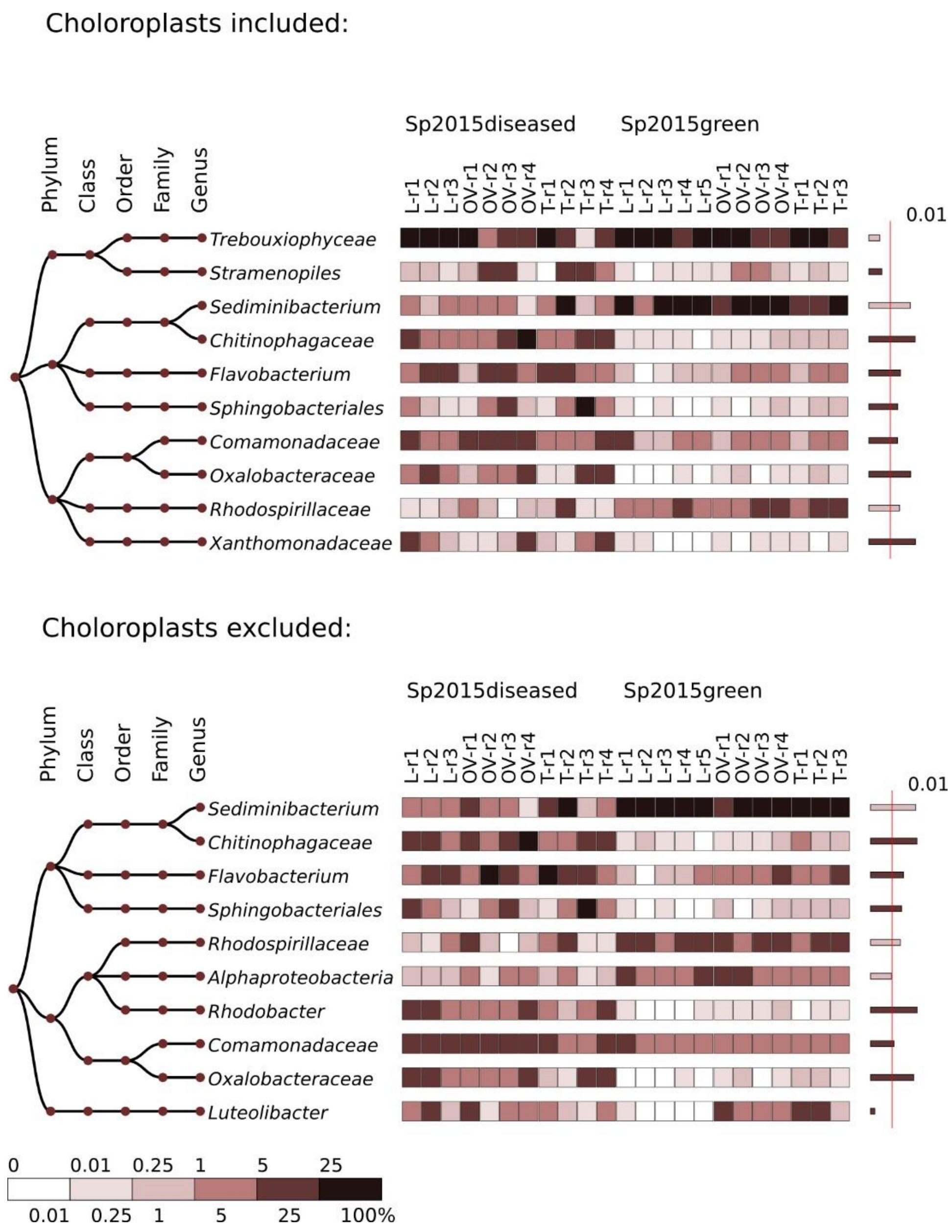

S6_fig. Heatmaps representing an effect of Chloroplast species on a separation of genera specific to diseased sponges. The columns on the right shows a significance difference between the healthy and diseased samples estimated using Mann-Whitney test; width of the bars corresponds to –ln (p-value). Red line separates the significance level of 0.99 (p-value < 0.01).

The analysis of third-party data of sponge microbiomes from coasts of New Guinea performed to support the methodology used to compare mixing sequencing technologies. The dataset was composed from raw sequencing reads deposited in projects PRJNA216132 (16S amplicon metagenome analysis of three sponge species at CO2 seeps in Papua New Guinea) and PRJNA454201 (Sponge microbiome

responses to ocean acidification) [Kander et al., 2018]. The data in project PRJNA216132, submitted by Australian Institute of Marine Sciences and RTL Genomics, was sequenced using 454 GS FLX+ pyrosequencer. 20 samples collected 27.08.2013 on control sites were used, png51c3-png60c3 for *Stylissa massa* sponge and png21c1-png30c1 for *Coelocarteria singapurensis* sponge. The data on project PRJNA454201, submitted by Victoria University of Wellington and Australian Centre for Ecogenomics, was sequenced in Illumina MiSeq with paired layout. Four samples collected 22.11.2014 on control sites were used, SC6.2, SC5.2 for *Stylissa flabelliformis* sponge and FC6.2, FC5.2 for *Coelocarteria singapurensis* sponge.

The 454 pyrosequencing reads were quallity filtered and trimmed using mothur package, with parameters 'maxambig=0, maxhomop=8, flip=T, bdiffs=1, pdiffs=2, qwindowaverage=25, qwindowsize=50, minlength=150'. The Illumina pair-end reads were quality trimmed using Trimmomatic (SLIDINGWINDOW:50:20 MINLEN:50) and merged using Flash software (-m 10 -x 0.2 -p 33 -r 300 -f 450 -s 150). The closed-reference OTU picking of the combined dataset was identical to the procedure applied to combined dataset of Baikal sponge samples, as it described in Methods section in the main text.

The *Stylissa* genus of sponges (Axinellidae) and *Coelocarteria singapurensis* sponges (Isodictyidae) are closer in microbiome composition than the *L. baikalensis* sponges in two states of disease. So, the variations between distance measures that separate two genera of sponges following same PERMANOVA approach with 4000 iterations observed on higher level of taxonomic hierarchy, as shown in S2 Table.

S2 Table. The results of PERMANOVA function for sea sponges.

Level	Pearson	Kendall	Spearman	Euclidean	Bray-Curtis	Jaccard	Morisita-Horn	UniFrac unweighted	UniFrac weighted
Family	0.00025	0.00025	0.0005	0.008	0.00025	0.00025	0.0005	0.00025	0.0005
Order	0.0085	0.00025	0.00625	0.01475	0.00025	0.00025	0.0105	0.00025	0.00025
Class	0.0845	0.00025	0.07675	0.02025	0.00075	0.00025	0.0795	0.00025	0.0045

The dendrograms constructed for presentation of dataset at order level using the same average linking and two measures of distance demonstrate that samples of both sponges' genera grouped together and the biases are the corrections of second order (S7_fig).

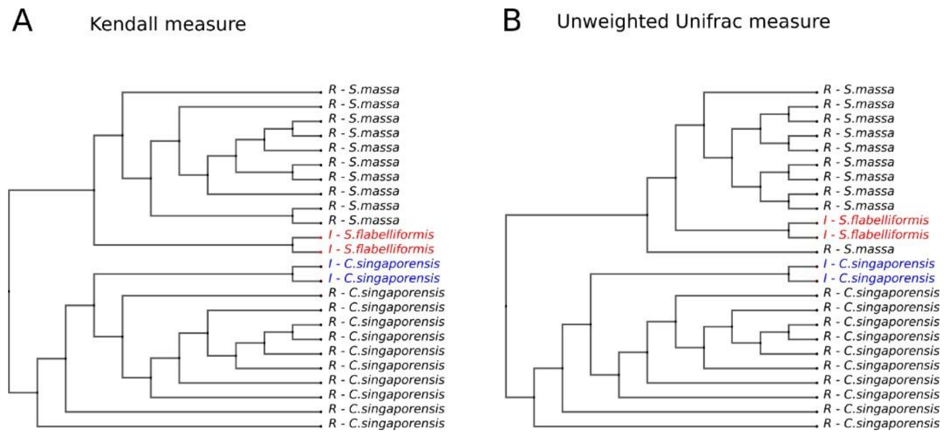

S7_fig. Dendrograms representing a degree of proximity between microbiome composition of two marine sponges. (A) Constructed using rank-based Kendall measure. (B) Presence-based unweighed UniFrac measure. Abundance values in samples used at level of family and dendrograms constructed using UPGMA clustering.

This could serve as additional support to the statement in the results section of the main text that samples of healthy *L. baicalensis* sponge from 2010 year separated from both samples of 2011 year and are close to some of healthy samples, collected in 2015 and sequenced using another technology.

In a similar way, the quantile normalization improves the separation of bacterial phylotypes specific to each of the sponge genera (S8_fig).

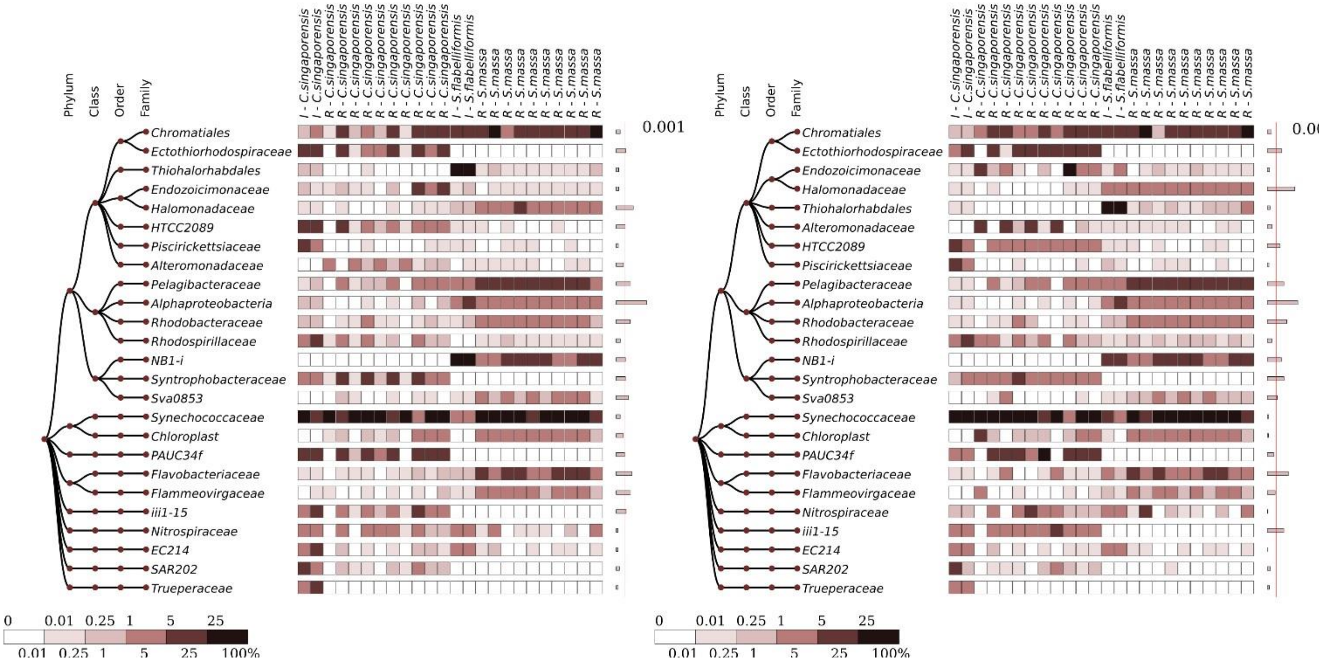

S8_fig. Heatmap of the 25 most abundant bacterial groups at the level of family. (A) Raw abundances. (B) After quantile normalization. The column on the right shows a significance difference between the two genera of host sponges, estimated using Mann-Whitney test; width of the bars corresponds to –ln (p-value). Red line separates the significance level of 0.999 (p-value < 0.001).

The values of alpha-diversity for the combined dataset of the marine sponges provided at level of order in taxonomy for reference (S3Table).

S3Table. The values of alpha-diversity for the combined dataset of the marine sponges.

SRA ID	Sample ID	Total	Unclassified	Ace	Chao1	Shannon	Simpson	OTU Number	Singletons	Doubletons
SRR957525	png21c1	5483	2	16.13	12	0.2	0.05	10	4	2
SRR957526	png22c1	1273	221	27.27	27	3.01	0.82	25	4	2
SRR957532	png23c1	2636	9	16.33	16	0.41	0.09	16	1	4
SRR957578	png24c1	2724	312	39.34	37.5	2.29	0.6	35	6	5
SRR957584	png25c1	1548	22	17.04	16.5	0.83	0.2	16	2	1
SRR957597	png26c1	1718	458	25.9	25	2.88	0.81	24	3	2
SRR957598	png27c1	2984	1	18.11	21	0.25	0.06	11	5	0
SRR957599	png28c1	1715	320	28.65	28.5	3.32	0.85	27	3	1
SRR957600	png29c1	2278	205	28.19	27.2	2.62	0.72	27	2	4
SRR957601	png30c1	1428	157	31.54	31.33	2.86	0.78	28	5	2
SRR958136	png51c3	5424	355	44.13	46	3.07	0.79	41	6	2
SRR958138	png52c3	10562	687	52.63	52.5	2.84	0.75	50	5	3
SRR958141	png53c3	3814	284	47.03	46.5	3.18	0.81	43	7	5
SRR958142	png54c3	10892	652	63.77	69.33	3.2	0.82	51	11	2
SRR958149	png55c3	3730	183	55.67	59.25	3.35	0.86	48	10	3
SRR958153	png56c3	2890	166	45.66	52	2.93	0.79	40	9	2
SRR958163	png57c3	4181	318	40.37	39.5	3.13	0.8	37	5	3
SRR958164	png58c3	105342	6891	96.41	94.5	3.24	0.81	90	9	7

SRR958165	png59c3	8230	531	56.33	55	3.15	0.81	49	9	5
SRR958166	png60c3	5498	161	38.85	39	2.22	0.6	37	4	2
SRR7081699	SC5.2	13118	609	109.2	112.25	2.3	0.71	49	23	3
SRR7081700	SC6.2	28300	1935	61.07	61	2.47	0.74	52	10	4
SRR7081710	FC5.2	15958	3145	52.2	46.75	4.11	0.93	43	6	3
SRR7081709	FC6.2	6694	1396	40.95	45	3.81	0.88	35	5	0

Details of implementation, fragments of code in Python 2.7 and bash provided to explain the features of downstream analysis.

Rarefaction and Michaelis-Menten fit:

```
#input: si - sorted list of abundance values
#output: xvals, yvals - rarefaction chart; mparams - parameters and precision of Michaelis-Menten fit

import skbio.stats as skstats
from scipy.optimize import fmin_powell

n_indiv = sum( si )
n_otu = len( si ) def
subsample( si, i ):
 ssi = skstats.subsample_counts( si, i )   return
np.count_nonzero( ssi ) def errfn(p, n, y):
        return ( ( ( p[0] * n / (p[1] + n ) ) - y ) ** 2 ).sum()
i_step = max( n_indiv / 200, 1 ) num_repeats = max(
2000 / i_step, 1 )
print >>sys.stderr, ( i_step, num_repeats )
S_max_guess = n_otu
B_guess = int( round( n_otu / 2 ) ) params_guess
= ( S_max_guess, B_guess ) xvals = np.arange(
1, n_indiv, i_step )
ymtx = np.empty( ( num_repeats, len( xvals ) ), dtype=int ) for
i in range( num_repeats ):
ymtx[i] = np.asarray( [ subsample( si, n ) for n in xvals ], dtype=int ) yvals
= ymtx.mean(0)
params_guess = ( n_otu,  int( round( n_otu / 2 ) ) )
mparams = fmin_powell( errfn, params_guess, ftol=1e-5, args=(xvals, yvals), disp = False )
```

Quantile normalization:

```
#input: matrix - matrix of relative abundances
#output: df - transformed matrix of relative abundances

import numpy as np

def quantileNormalize( matrix ):
    df = copy.copy( matrix )
    dic = {}     for col in range( len( df )
):         dic.update({col :
sorted(df[col])})     rank = [ 0 ] * len(
df[0] )     for i in range( len( rank ) ):
rank[ i ] = np.average( [ dic[ col ][ i ] for col in range( len( df ) ) ] )     for col in range(
len( df ) ):         t = np.searchsorted( np.sort(df[col]), df[col] )         df[col] = [ rank[i]
for i in t ]
    return df
```

Reduction of dataset:

```
#input: data (double list of strings), ml (integer) - representation of raw data on OTU level; ilevel - level of reduction in taxonomic hierarchy;
kdict - taxonomic assignments in that level;  findex (list of intergers), gtags (distionary) - pre-defined distribution of samples into groups;
#output: edata - matrix of absolute abundance counts, distributed to groups and in a reduced level of taxonomy
 def load_edata( data, ilevel, ml, kdict, findex, gtags
):
            edata = []                for tagnum in sorted( gtags.values() ):
cgtag = gtags.keys()[ gtags.values().index( tagnum ) ]
                        crow = [ 0 ] * len( kdict )                        for i in sorted( findex ):
                        if findex[i] == cgtag:                                            for d
in data[1:]:                                                            if len( d ) < max(
findex.keys() ) + ml:
                                                                        continue
    ckey = "".join( d[0:ilevel] )      if ckey in kdict:
                                                                        cind = kdict[ ckey ]
                                                                        v = int( float( d[ i + ml + 1 ] ) )
                                                                        crow[ cind ] += v
                        edata.append( crow )
            return edata
```

Selection of most abundant phylotypes:

```
#input: edata - matrix of absolute abundance counts; num_best - number of taxonomic units to select; kdict - taxonomic assignents for rows
in input matrix
#output: nedata - matrix of absolute abundance counts for num_best most abundant units; nkdict - taxonomic assignents for the selected
abundant units

import numpy as np
```

```
 def select_toptax( edata, kdict, num_best ):     aedata = np.array( edata, dtype=float )
    aenorm = np.maximum( np.sum( aedata, axis=1 ), np.array( [ 1. / len( edata[0] ) ] * len( edata ) ) )
aedata /= aenorm.reshape( len(edata), 1 )     ssum = np.sum( aedata, axis=0 )
    ssorted = sorted( ssum.tolist(), reverse=True )
smax = ssorted[ num_best ]     nkdict = {}     nedata =
[]     tcnt = 0     for key in kdict:         if ssum[ kdict[key]
] > smax and tcnt < num_best:                   for k in
range( len( edata ) ):               if tcnt == 0:
                    nedata.append( [] )
nedata[k].append( edata[k][ kdict[key] ] )
                nkdict[ key ] = tcnt
tcnt += 1
    return ( nedata, nkdict )
```

<u>Functional annotation (fragment of script in bash):</u>

```
source activate qiime1
filter_samples_from_otu_table.py -i otu_table.biom -o picrust_input.biom -n 1
normalize_by_copy_number.py -i picrust_input.biom -o normalized_otus.biom
predict_metagenomes.py -i normalized_otus.biom -o picrust.biom
```
