# Supplementary figures and images for: Diversity and shifts of the bacterial community associated with Baikal sponge mass mortalities
Source: PLoS One. 2019 Mar 28;14(3):e0213926. doi: 10.1371/journal.pone.0213926 (PMC6438488; doi:10.1371/journal.pone.0213926)

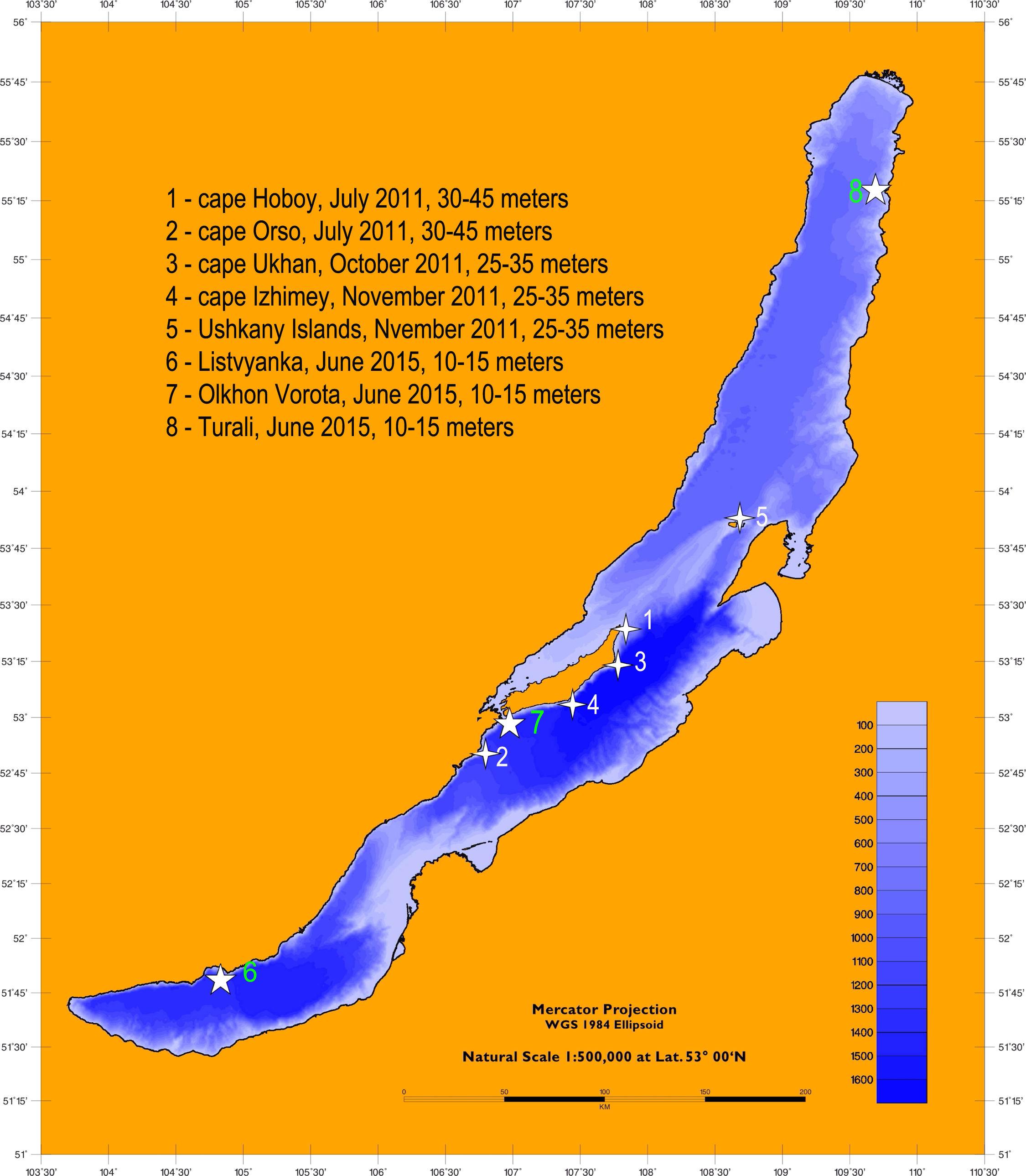

Supplement: S1 Fig — Four pointed stars—places of detection of pink sponges in 2011; 5 pointed stars—places for collecting samples of healthy and sick sponges in 2015. 1—cape Hoboy, July 2011, 30–45 meters; 2 –cape Orso, July 2011, 30–45 meters; 3 –cape Ukhan, October 2011, 25–35 meters; 4– cape Izhimey, November 2011, 45–55 meters; 5 –Ushkany Islands, November 2011, 25–35 meters. (TIF) [file pone.0213926.s003.tif]

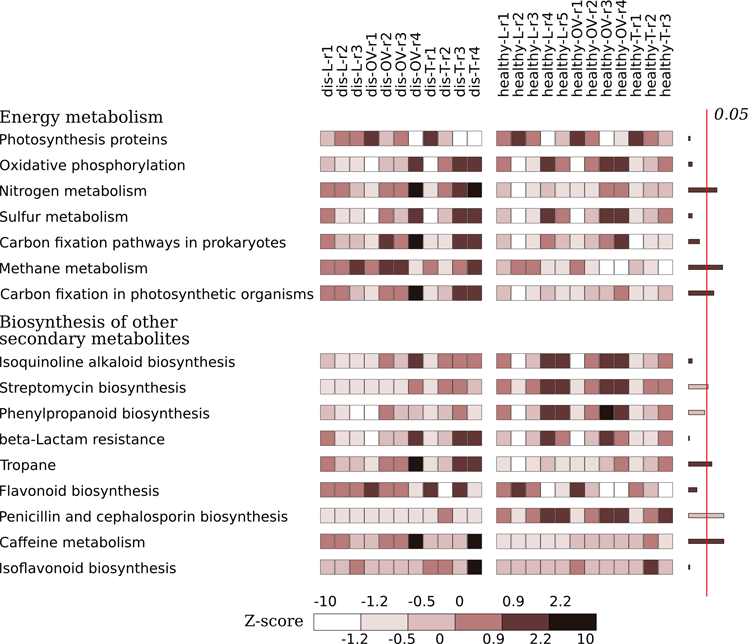

Supplement: S2 Fig — The relative presence of functional groups in microbiomes of sponges from 2015 are presented as heatmap chart, in units of Z-score, for 'Energy metabolism' and 'Biosynthesis of Other Secondary Metabolites' KEGG ontology terms. (TIF) [file pone.0213926.s004.tif]
