# Supplementary material for: Diversity and shifts of the bacterial community associated with Baikal sponge mass mortalities
Source: PLoS One. 2019 Mar 28;14(3):e0213926. doi: 10.1371/journal.pone.0213926 (PMC6438488; doi:10.1371/journal.pone.0213926)
Supplement: S2 Table — (PDF) [file pone.0213926.s006.pdf]

S2 Table. **Values for alpha-diversity and estimates of significance for differences between diversity indices.**

Supplementary material to the study

Diversity and shifts of the bacterial community associated with Baikal sponge mass mortalities

authored by

Sergei Belikov, Natalia Belkova, Tatiana Butina, Lubov Chernogor, Alexandra Martynova-Van Kley, Armen Nalian, Colin Rorex, Igor Khanaev, Olga Maikova, Sergey Feranchuk

1. Alpha-diversity:

1.1 Level of Family expanded:

1.1a Chloroplast included:

Illumina:

*	Total	Unclassified	Ace	Chao1	Shannon	Simpson	OTU Number	Michaelis-Menten	Singletons	Doubletons
Sp2011pink 357	12079	599	65.73	64	2.54	0.67	61	62.27	7	6
Sp2011pink 515	11412	536	54	54	2.54	0.67	54	55.81	0	7
Sp2010healthy 357	61354	4357	58.77	58.25	2.33	0.64	58	58.51	2	3
Sp2010healthy 515	57440	4130	59.03	61	2.36	0.65	58	58.34	3	0
Sp2011green 357	49710	1777	55.87	54.86	1.98	0.58	54	56.02	4	6
Sp2011green 515	47067	1699	50.3	50	2.01	0.59	50	51.86	1	7

454:

*	Total	Unclassified	Ace	Chao1	Shannon	Simpson	OTU Number	Michaelis-Menten	Singletons	Doubletons
Sp2011diseased L-r1	1518	116	49.22	46.36	3.67	0.86	45	49.15	6	10
Sp2011diseased L-r2	2007	76	47.79	46	2.97	0.74	42	45.68	8	6
Sp2011diseased L-r3	2032	67	64.51	60.6	3.04	0.7	54	59.43	12	9
Sp2011diseased OV-r1	2806	98	58.38	55.6	2.24	0.56	49	56.14	12	9
Sp2011diseased OV-r2	1379	211	53.76	43.17	3.32	0.86	34	36.49	11	5
Sp2011diseased OV-r3	1059	363	47.59	44.14	3.87	0.91	39	42.1	9	6
Sp2011diseased OV-r4	2914	228	63.85	56.25	3.58	0.86	48	50.24	12	7
Sp2011diseased T-r1	4545	40	63.85	62.33	2.22	0.58	55	62.13	12	8
Sp2011diseased T-r2	2245	307	93.79	98	3.91	0.87	77	89.53	22	10
Sp2011diseased T-r3	2953	1274	63.34	79	3.31	0.85	44	47.32	15	2
Sp2011diseased T-r4	3283	475	94.64	94.1	4.51	0.93	85	92.31	14	9
Sp2015green L-r1	4122	232	92.34	78.3	2.17	0.67	53	67.87	23	9
Sp2015green L-r2	4579	35	28.82	29.25	0.56	0.14	24	29.08	7	3
Sp2015green L-r3	4788	92	67.93	59.91	1.59	0.56	49	61.55	16	10
Sp2015green L-r4	2591	67	42.41	42.25	1.76	0.53	31	35.47	10	3
Sp2015green L-r5	4067	184	53.86	47.14	1.78	0.59	36	44.42	13	6
Sp2015green OV-r1	3807	238	46.04	47.25	1.83	0.45	42	45.81	7	3
Sp2015green OV-r2	2251	187	54.12	55	2.55	0.7	44	48.82	12	5
Sp2015green OV-r3	2168	177	61.6	71.25	2.3	0.59	45	51.26	15	3
Sp2015green OV-r4	1957	168	67.76	64.5	2.93	0.75	47	52.73	15	5
Sp2015green T-r1	4624	133	101.08	105.3	2.22	0.54	80	93.44	23	9
Sp2015green T-r2	1868	101	73.51	74.5	3.16	0.8	49	54.74	18	5
Sp2015green T-r3	5385	293	100.5	89.1	3.2	0.81	66	73.03	22	9

1.1b Chloroplast excluded:

Illumina:

*	Total	Unclassified	Ace	Chao1	Shannon	Simpson	OTU Number	Michaelis-Menten	Singletons	Doubletons
Sp2011pink 357	12052	589	61.25	60.14	2.52	0.67	58	62.27	6	6
Sp2011pink 515	11386	527	52	52	2.52	0.66	52	55.81	0	7
Sp2010healthy 357	26890	3825	53.81	53.25	2.88	0.74	53	58.51	2	3
Sp2010healthy 515	25344	3577	54.12	56	2.92	0.74	53	58.34	3	0
Sp2011green 357	18756	1605	50.24	49.5	2.62	0.75	49	56.02	3	5
Sp2011green 515	18081	1540	46.32	46	2.63	0.75	46	51.86	1	7

454:

*	Total	Unclassified	Ace	Chao1	Shannon	Simpson	OTU Number	Michaelis-Menten	Singletons	Doubletons
Sp2011diseased L-r1	1044	110	45.47	42.91	3.98	0.91	42	45.99	5	10
Sp2011diseased L-r2	1028	62	45.79	44	3.75	0.89	40	44.12	8	6
Sp2011diseased L-r3	954	62	61.81	58.33	4.3	0.92	51	55.96	12	8
Sp2011diseased OV-r1	964	80	56.38	53.6	3.67	0.87	47	54.89	12	9
Sp2011diseased OV-r2	1168	34	51.76	41.17	3.07	0.83	32	35.4	11	5
Sp2011diseased OV-r3	796	189	45.59	42.14	3.76	0.9	37	40.87	9	6
Sp2011diseased OV-r4	2704	224	63.38	54.25	3.45	0.85	46	48.29	12	7
Sp2011diseased T-r1	1700	40	62.85	61.33	3.38	0.81	54	61.51	12	8
Sp2011diseased T-r2	1830	134	89.93	93	3.67	0.83	72	85.45	22	10
Sp2011diseased T-r3	2710	1037	63.75	76	3.13	0.82	41	44.37	15	2
Sp2011diseased T-r4	2879	357	92.64	92.1	4.41	0.92	83	90.95	14	9
Sp2015green L-r1	2422	220	89.33	78.11	1.98	0.54	50	65.87	23	8
Sp2015green L-r2	343	33	23.7	23.5	2.29	0.63	21	25.59	5	3
Sp2015green L-r3	2154	81	66.4	57.33	1.26	0.29	44	55.39	16	8
Sp2015green L-r4	2080	60	38.26	36	1.27	0.34	27	30.65	9	3
Sp2015green L-r5	2833	180	49.54	42.43	1.27	0.35	33	41.23	12	6
Sp2015green OV-r1	1002	231	44.25	45.25	3.74	0.88	40	43.92	7	3
Sp2015green OV-r2	1330	156	52.12	53	2.52	0.6	42	47.79	12	5
Sp2015green OV-r3	1844	153	56.68	64.75	1.92	0.47	42	48.9	14	3

Sp2015green OV-r4	1591	152	65.46	64	2.67	0.66	43	48.53	15	4
Sp2015green T-r1	1543	123	94.43	97	3.83	0.84	76	89.09	21	9
Sp2015green T-r2	1164	83	73.89	74.6	3.4	0.83	44	49.07	18	4
Sp2015green T-r3	4542	250	96.51	86.67	2.98	0.76	61	68.78	22	8

1.2 Shannon index, all levels (chloroplasts included):

*	OTU	Species	Genus	Family	Order	Class	Phylum
Sp2011pink 357	3.42	2.59	2.59	2.54	2.45	2.34	1.83
Sp2011pink 515	3.49	2.58	2.58	2.54	2.45	2.33	1.82
Sp2011diseased L-r1	5.09	4.01	3.94	3.67	3.37	2.81	1.67
Sp2011diseased L-r2	4.3	3.25	3.17	2.97	2.69	2.38	1.67
Sp2011diseased L-r3	3.98	3.28	3.22	3.04	2.88	2.45	1.6
Sp2011diseased OV-r1	2.8	2.34	2.33	2.24	2.14	1.92	1.25
Sp2011diseased OV-r2	5.5	3.61	3.49	3.32	2.73	2.53	1.46
Sp2011diseased OV-r3	6.22	4.23	4.18	3.87	3.63	3.1	1.7
Sp2011diseased OV-r4	5.38	3.76	3.72	3.58	3.15	2.74	1.6
Sp2011diseased T-r1	2.99	2.38	2.36	2.22	2.14	2.01	1.42
Sp2011diseased T-r2	5.51	4.19	4.15	3.91	3.54	2.98	1.86
Sp2011diseased T-r3	4.89	3.47	3.44	3.31	3.06	2.94	1.35
Sp2011diseased T-r4	6.68	4.86	4.83	4.51	4.02	3.31	1.93
Sp2010healthy 357	2.56	2.35	2.35	2.33	2.26	2.04	1.72
Sp2010healthy 515	2.6	2.39	2.39	2.36	2.3	2.07	1.73
Sp2011green 357	2.34	1.99	1.99	1.98	1.96	1.87	1.23
Sp2011green 515	2.38	2.01	2.01	2.01	1.98	1.9	1.25
Sp2015green L-r1	2.4	2.19	2.19	2.17	2.11	1.9	1.59
Sp2015green L-r2	0.65	0.56	0.56	0.56	0.55	0.52	0.49
Sp2015green L-r3	1.73	1.6	1.6	1.59	1.56	1.47	1.35
Sp2015green L-r4	1.98	1.78	1.78	1.76	1.7	1.57	1.33
Sp2015green L-r5	1.94	1.8	1.79	1.78	1.74	1.62	1.42
Sp2015green OV-r1	2.08	1.85	1.85	1.83	1.76	1.56	1.31
Sp2015green OV-r2	3.02	2.62	2.61	2.55	2.45	2.16	1.83
Sp2015green OV-r3	2.68	2.35	2.35	2.3	2.23	1.99	1.6

Sp2015green OV-r4	3.69	3.03	3.03	2.93	2.77	2.57	1.93
Sp2015green T-r1	2.69	2.31	2.31	2.22	2.11	1.93	1.43
Sp2015green T-r2	3.94	3.27	3.27	3.16	3.02	2.81	2.07
Sp2015green T-r3	4.15	3.29	3.29	3.2	3.03	2.74	1.88

2. Significance of separation between subsets of samples, in units of p-value

2.1 Chloroplasts included:

2.1.1 healthy 2010 + healthy2011 + diseased2011 + healthy2015 + diseased2015 / anova

**Level**	Ace	Chao1	Shannon	Simpson	OTU Number
OTU	0.033	0.041	0	0.026	0.015
Species	0.64	0.602	0.002	0.041	0.332
Genus	0.693	0.691	0.003	0.044	0.428
Family	0.911	0.955	0.005	0.046	0.771
Order	0.95	0.975	0.014	0.077	0.953
Class	0.995	0.985	0.026	0.095	0.646
Phylum	0.819	0.877	0.378	0.324	0.746

2.1.2 healthy2015 + diseased2015 / ranked test

**Level**	Ace	Chao1	Shannon	Simpson	OTU Number
OTU	0.005	0.005	0	0.002	0.002
Species	0.104	0.186	0	0.003	0.012
Genus	0.17	0.221	0.001	0.003	0.018
Family	0.391	0.415	0.001	0.003	0.269
Order	0.439	0.391	0.002	0.004	0.379
Class	0.488	0.403	0.003	0.005	0.239
Phylum	0.24	0.239	0.322	0.322	0.426

2.1.3 2015 Listvyanka + Olkhon Vorota + Turali / anova

Level	Ace	Chao1	Shannon	Simpson	OTU Number
OTU	0.001	0	0.114	0.171	0.003
Species	0.027	0.002	0.114	0.201	0.004
Genus	0.033	0.002	0.099	0.198	0.003
Family	0.003	0	0.087	0.203	0.001
Order	0.001	0.001	0.076	0.208	0.001
Class	0.001	0.001	0.04	0.197	0.002
Phylum	0.099	0.035	0.172	0.537	0.009

2.2 Chloroplasts excluded:

2.2.1 healthy 2010 + healthy2011 + diseased2011 + healthy2015 + diseased2015 / anova:

Level	Ace	Chao1	Shannon	Simpson	OTU Number
OTU	0.038	0.052	0	0.001	0.019
Species	0.558	0.538	0.001	0.003	0.263
Genus	0.617	0.629	0.001	0.003	0.35
Family	0.849	0.923	0.004	0.004	0.724
Order	0.948	0.983	0.02	0.012	0.957
Class	0.995	0.985	0.021	0.008	0.646
Phylum	0.819	0.877	0.378	0.324	0.746

2.2.2 healthy2015 + diseased2015 / ranked test:

Level	Ace	Chao1	Shannon	Simpson	OTU Number
OTU	0.004	0.005	0	0	0.002
Species	0.141	0.186	0	0	0.012

Genus	0.155	0.221	0	0	0.013
Family	0.415	0.439	0.001	0.001	0.23
Order	0.463	0.488	0.004	0.002	0.463
Class	0.488	0.403	0.004	0.001	0.239
Phylum	0.24	0.239	0.322	0.322	0.426

2.2.3 2015 Listvyanka + OlkhonGate + Turali / anova:

**Level**	Ace	Chao1	Shannon	Simpson	OTU Number
OTU	0.001	0	0.158	0.067	0.004
Species	0.026	0.002	0.169	0.093	0.006
Genus	0.033	0.003	0.137	0.085	0.004
Family	0.002	0	0.113	0.088	0.001
Order	0	0	0.095	0.082	0.001
Class	0.001	0.001	0.016	0.04	0.002
Phylum	0.099	0.035	0.172	0.537	0.009
